# Identification of QTL with large effect on seed weight in a selective population of soybean with genome-wide association and fixation index analyses

**DOI:** 10.1186/s12864-017-3922-0

**Published:** 2017-07-12

**Authors:** Long Yan, Nicolle Hofmann, Shuxian Li, Marcio Elias Ferreira, Baohua Song, Guoliang Jiang, Shuxin Ren, Charles Quigley, Edward Fickus, Perry Cregan, Qijian Song

**Affiliations:** 1 Institute of Cereal and Oil Crops, Hebei Academy of Agricultural and Forestry Sciences/ Shijiazhuang Branch of National Soybean Improvement Center / Key Laboratory of Crop Genetics and Breeding of Hebei, Shijiazhuang, 050035 China; 2 Soybean Genomics and Improvement Laboratory, United States Department of Agriculture, Agricultural Research Service, 10300 Baltimore Ave, Building 006, Beltsville, MD 20705 USA; 3 United States Department of Agriculture, Agricultural Research Service (USDA-ARS), Crop Genetics Research Unit, Stoneville, MS 38776 USA; 4 EMBRAPA Genetic Resources and Biotechnology, Embrapa, Brasília, DF C.P.02372 Brazil; 5 Department of Biological Sciences, University of North Carolina at Charlotte, Charlotte, NC 28223 USA; 6 Agricultural Research Station, Virginia State University, P.O. Box 9061, Petersburg, VA 23806 USA; 7 Present address: Davare Laboratory, Pediatric Cancer Biology Program, Oregon Health and Science University, 3181 SW Sam Jackson Park Rd, Portland, OR 97239 USA

**Keywords:** Soybean, Selective population, Seed weight, GWAS, SNP, Fixation index analysis

## Abstract

**Background:**

Soybean seed weight is not only a yield component, but also a critical trait for various soybean food products such as sprouts, edamame, soy nuts, natto and miso. Linkage analysis and genome-wide association study (GWAS) are two complementary and powerful tools to connect phenotypic differences to the underlying contributing loci. Linkage analysis is based on progeny derived from two parents, given sufficient sample size and biological replication, it usually has high statistical power to map alleles with relatively small effect on phenotype, however, linkage analysis of the bi-parental population can’t detect quantitative trait loci (QTL) that are fixed in the two parents. Because of the small seed weight difference between the two parents in most families of previous studies, these populations are not suitable to detect QTL that have considerable effects on seed weight. GWAS is based on unrelated individuals to detect alleles associated with the trait under investigation. The ability of GWAS to capture major seed weight QTL depends on the frequency of the accessions with small and large seed weight in the population being investigated. Our objective was to identify QTL that had a pronounced effect on seed weight using a selective population of soybean germplasm accessions and the approach of GWAS and fixation index analysis.

**Results:**

We selected 166 accessions from the USDA Soybean Germplasm Collection with either large or small seed weight and could typically grow in the same location. The accessions were evaluated for seed weight in the field for two years and genotyped with the SoySNP50K BeadChip containing >42,000 SNPs. Of the 17 SNPs on six chromosomes that were significantly associated with seed weight in two years based on a GWAS of the selective population, eight on chromosome 4 or chromosome 17 had significant Fst values between the large and small seed weight sub-populations. The seed weight difference of the two alleles of these eight significant SNPs varied from 8.1 g to 11.7 g/100 seeds in two years. We also identified haplotypes in three haplotype blocks with significant effects on seed weight. These findings were validated in a panel with 3753 accessions from the USDA Soybean Germplasm Collection.

**Conclusion:**

This study highlighted the usefulness of selective genotyping populations coupled with GWAS and fixation index analysis for the identification of QTL with substantial effects on seed weight in soybean. This approach may help geneticists and breeders to more efficiently identify major QTL controlling other traits. The major regions and haplotypes we have identified that control seed weight differences in soybean will facilitate the identification of genes regulating this important trait.

**Electronic supplementary material:**

The online version of this article (doi:10.1186/s12864-017-3922-0) contains supplementary material, which is available to authorized users.

## Background

Soybean [*Glycine max* (L.) Merr.] is one of the most important crops worldwide. It accounts for about 68% of the world protein meal and 57% of the world vegetable oil production. Soybean seed weight is not only a yield component, but also a critical trait for various soybean food products such as sprouts, edamame, soy nuts, natto and miso [1]. Soybean seed weight is a complex trait affected by many genetic and environmental factors such as temperature and precipitation during the seed development stage. The heritability of soybean seed weight has been reported to range from 44 to 94% [2].

Linkage analysis and genome-wide association studies (GWAS) are two complementary and powerful tools to connect phenotypic differences to the underlying causative loci. Previous studies have identified quantitative trait loci (QTL) associated with seed weight in soybean recombinant inbred lines (RILs) and these QTL are documented in SoyBase [3]. This information will help breeders and geneticists to understand the genetic architecture underlying seed weight. Since linkage analysis is based on progeny derived from two parents, linkage analysis of the bi-parental population can’t detect QTL that are fixed in the two parents. As the number of recombination events in each soybean RIL is limited [4], resolution of the QTL position on the linkage map is frequently low. There were caveats in the previous soybean seed weight reports, e. g., some of the RIL populations were developed for the detection of QTL for other traits, not specifically for seed weight. Among 24 *G.max* × *G.max* RIL populations used for the detection of seed weight QTL in the past decades as described in SoyBase [3], a total of 16 populations had seed weight differences of less than 5.0 g/100 seeds between the two parents used in each cross. Thus, subsequent analysis of these populations was highly unlikely to identify QTL with substantial effects. Also, it was unlikely to detect QTL correctly due to experimental effects. Even though some QTL have been identified, it has been difficult to distinguish QTL with major or minor effects.

GWAS is based on population samples of unrelated individuals to detect alleles associated with a trait under study and has been widely used to detect agronomic, seed composition and disease resistance QTL in soybean [1, 3, 5–11]. Several GWAS were conducted to identify seed weight QTL based on the single nucleotide polymorphism (SNP) BeadChip Assay or genome re-sequencing. Zhang et al. [1] conducted GWAS of seed weight in a population with 309 soybean accessions from Maturity Groups 0 and 00. The seed weight for most accessions was between 13.1–17.1 g. In another study, Zhang et al. [10] analyzed 366 landrace accessions that were native to six soybean eco-regions in China, but only 41 accessions had 100 seed weights larger than 20 g. Zhou et al. [11] randomly selected 286 soybean accessions including 14 wild, 153 landrace, and 119 derived from breeding programs from six geographic regions in China for the detection of seed weight QTL. However, the low frequency of QTL alleles with substantial effect in these association panels may limit the power of GWAS [12] since the effects of such low frequency alleles may not be captured. Bandillo et al. [13] reported that if the frequency of the seed protein-enhancing QTL alleles was lower than the minor allele frequency (MAF) threshold of GWAS, the QTL that were previously identified and confirmed in bi-parental QTL mapping populations could not be detected by GWAS. In addition, as most previously studied association populations included accessions from a wide range of maturity groups, these accessions may not be well adapted to the same growing environment and would result in immature or abnormal seeds for seed weight measurement.

Selective genotyping is a term first used by Lander and Botstein [14] to describe studies that only individuals from the high and low extremes of the trait distribution are selected to test the association of traits with markers. The value of selective genotyping for GWAS has been widely recognized in human association studies [15–17], e.g. case and control is the commonly used design to include only the two types of extreme individuals in human epidemiology studies and clinic trials. The method is very effective for screening a large number of potential donors for large-effect QTL alleles governing a particular trait of interest [12, 18]. Simulation showed that for a fixed number of phenotypic individuals this approach increased power relative to random sampling and may reduce statistical error [18, 19]. The approach was considered as an efficient alternative to the analysis of the entire population in linkage analysis and GWAS [20], and has been used to identify genes controlling various traits in a number of bi-parental segregating populations of species such as barley, rice and soybean [12, 18, 20]. Nonetheless the creation of association panels using selective genotyping is very limited in plants.

In this study, we assembled a selective population containing a high frequency of soybean accessions with large and small seed weight from Maturity Groups II, III and IV, examined their allelic differences and conducted a GWAS and fixation index analysis of seed weight in the population. The objective was to identify candidate QTL that had pronounced effects on soybean seed weight.

## Methods

### Plant materials and field trials

One hundred and sixty-six soybean Plant Introductions (PIs) from Maturity Groups II, III and IV were obtained from the USDA Soybean Germplasm Collection (Urbana, IL) (Additional file 1: Table S1). These maturity groups were chosen because their photoperiod response allows them to mature appropriately in Beltsville, Maryland. The collection consisted of 85 PIs with small 100-seed weight, ranging from 4.2 g to 10.0 g, and 81 PIs with large seed weight, ranging from 20.0 g to 38.0 g, based on the information supplied by the Germplasm Resources Information Network (GRIN) (http://www.ars-grin.gov/). The two groups were carefully balanced in terms of Asian country of origin, maturity group, stem growth habit and flower color. Two replications of a randomized block design with the large seed and small seed weight sub-populations were planted in 2012 and 2013 at the USDA-ARS farms in Beltsville, Maryland. The seeds were harvested at full maturity, and a sample of 100 cleaned seeds from each plot was randomly selected and weighed.

### Genotyping and quality control

The Illumina Infinium SoySNP50K BeadChip containing 52,000 SNPs was used to genotype the population as described by Song et al. [21, 22]. SNPs present in unanchored sequence scaffolds or with MAF < 5% in the population were excluded.

### Linkage disequilibrium (LD) estimation

Pair-wise LD (*r* ^*2*^) between SNPs was calculated using the TASSEL program with an LD window size of 100 SNPs [23]. Only *r* ^*2*^ for SNPs with pairwise physical distance less than 10 Mb were used to determine the average LD decay. LD was estimated separately for euchromatic and heterochromatic regions of each chromosome due to the substantial difference in recombination rate between the two regions. Haploview 4.2 was used to determine haplotype blocks based on the confidence interval method [24].

### Population structure

A total of 7244 SNPs with LD less than 0.50 to adjacent loci were selected using the program PLINK (Version 1.07) [25] and were used to examine the population structure of the 166 accessions using STRUCTURE 2.3.4 [26]. The number of subsets (*k*) was tested from 2 to 10, and the burn-in time and iterations for each run were both set to 100,000. Five runs were used for each *k*. Ln P (D) and Evanno’s Δ*k* was used to determine the most appropriate *k* value, where Δ*k* = *M*[|L(k – 1) – 2 L(*k*) + L(*k* + 1)|]/*S*[L(*k*)], and L(*k*) represents the *k*th Ln P (D), M was the mean of 5 runs, and S was their standard deviation [27]. The unweighted pair group with arithmetic mean (UPGMA) dendrogram was constructed based on the p-distance of SNPs using the software Mega 7.0 [28].

### Statistical analysis

In order to obtain variance components for the calculation of heritability of seed weight, an analysis of variance was performed using the general linear model procedure of SAS version 9.3 [29]. The model for the phenotypic trait was *y*_*ijk*_ = *μ* + *g*_*i*_ + *l*_*j*_ + *(gl)*_*ij*_ + *e*_*ijk*_, where *μ* is the overall mean, *g*_*i*_ is the genetic effect of the *i* ^*th*^ genotype, *l*_*j*_ is the effect of the *j* ^*th*^ environment (year), *(gl)*_*ij*_ is the interaction effect between the *i* ^*th*^ genotype and the *j* ^*th*^ environment (year), and *e*_*ijk*_ is a random error following N(0,*σ* ^*2*^_*e*_). The heritability of seed weight was calculated as *H* ^2^ = *σ* ^*2*^_*g*_/[*σ* ^*2*^_*g*_ + σ^2^_*gl*_/*k* + σ^2^_*e*_/(*rk*)], where *σ* ^*2*^_*g*_ is the genotypic variance, *σ* ^*2*^_*gl*_ is the genotype x environment (year) interaction variance, *k* is the number of environments (years) and *r* is the number of replications [30]. In order to measure the population differentiation between the large and small seed weight sub-populations, the fixation index (Fst) was calculated for each locus using Arlequin v3.1 [31]. The mixed linear model (MLM) accounting for both population structure and kinship was conducted for genome-wide association analysis using the TASSEL program [23]. A value of -log (p) >3.0, where the p is the significance level of each locus, was obtained from TASSEL, and was used as a threshold to identify the QTL. The MLM can be expressed as *y* = *μ* + *Xα* + *Pβ* + *Zu* + *e*, where y is the vector of phenotypic observations, *μ* is the overall mean, *α* is the vector of SNP effects, *X* is the incidence matrix relating the individuals to the fixed marker effects *α*, *β* is the vector of population structure effect, *P* is the incidence matrix relating the individuals to the fixed population structure effects *β*, *u* is the vector of kinship background effects, *Z* is the incidence matrix relating the individuals to the random group effects *u* and *e* is the vector of residual effects [32]. The kinship coefficient matrix that explained the most probable identity by state of each allele between individuals was estimated using the TASSEL program [23]. The seed weight difference among haplotypes of each haplotype block was compared using SAS version 9.3 [29].

### Validation of haplotype association with seed weight in the 3753 accessions in the USDA soybean germplasm collection

A total of 3753 accessions in the USDA Soybean Germplasm Collection with seed weight greater than 20 g (1936 accessions) or smaller than 10 g (1817 accessions) were obtained from the USDA-ARS GRIN site (http://www.ars-grin.gov/). In order to validate the association of the seed weight with the haplotypes observed in the 166 PIs, the seed weight among haplotypes of 3753 accessions in the USDA Soybean Germplasm Collection was compared in each haplotype block using the analysis of variance procedure of SAS version 9.3 [29]. The 3753 accessions have been genotyped with the SoySNP50K Beadchip as previously described [21, 22].

### Permutation test of the association of Fst level with proportion of explained variance and allelic effects of the SNPs

To examine if the Fst analysis with the GWAS in the selective population can facilitate the identification of the SNPs with pronounced effects, a total of 20 sets of samples were randomly selected from the USDA Soybean Germplasm Collection with seed weight greater than 20 g or smaller than 10 g for GWAS and Fst analysis. Each set consisted of 166 accessions including 77 large, 85 small seed weight and four medium seed weight. A permutation test based on the SNP alleles of the 166 accessions in the SoySNP50K BeadChips dataset [22] and the seed weight of the 166 accessions was performed. The correlation coefficient of Fst with proportion of variance and allelic effects of the SNPs were estimated.

## Results

### Distribution of seed weight in the selective population

The distribution of seed weight for 166 soybean accessions showed two distinct peaks each year (Additional file 2: Figure S1), corresponding to sub-populations of small and large seed weight. The large seed weight sub-population consisted of 77 accessions with 100-seed weight varying from 19.2 g to 35.8 g, an average 100-seed weight of 24.8 g was calculated based on the mean observed in 2012 and 2013. The small seed weight population was composed of 85 accessions with 100-seed weight varying from 6.2 g to 14.6 g and averaged 10.0 g in 2012 and 2013. There were four accessions that could not be unambiguously placed in either large or small seed weight sub-populations due to large seed weight differences between the two years. The correlation of the seed weight was *r* = 0.946 between the values from GRIN and year 2012 and *r* = 0.948 between the GRIN values and year 2013. Variance analysis indicated that effects of genotype, year and genotype × year were significant (Additional file 3: Table S2). The broad-sense heritability of seed weight was 0.98 in the analysis of the two years of field experiments. The phenotypic correlation of the seed weight between the two years was high and significant (*r* = 0.971).

### Distribution of SNPs and extent of LD

DNA genotyping with the Illumina Infinium SoySNP50K BeadChip provided 42,509 high quality SNPs with a call success rate of >85%. A total of 35,009 SNPs with MAF ≥ 0.05 or missing and ambiguous alleles <0.15 was used for analysis (Table 1). These SNPs were generally evenly selected from euchromatic and heterochromatic regions of the 20 soybean chromosomes. A total of 29,819 SNPs were located in euchromatic regions with a marker density of 15.4 kb per SNP, while 5190 SNPs were located in heterochromatic regions with a marker density of 102.9 kb per SNP. In euchromatic regions, the mean value of LD measured by *r* ^*2*^ dropped to half its maximum value at an average distance of 122 kbp, while in heterochromatic regions, it reached half its maximum value at 1225 kbp (Additional file 4: Figure S2).

**Table 1:** Number and density of SNPs in euchromatic (EU) and heterochromatic (HET) regions, sequence length, and the average SNP density of each chromosome

Chromosome	Number of SNPs in EU regions	Sequence length of EU regions (Mb)	SNP density in EU regions (Kb/SNP)	Number of SNPs in HET regions	Sequence length of HET regions (Mb)	SNP density in HET regions (Kb/SNP)	Number of SNPs	Sequence length (Mb)	SNP density (Kb/SNP)
Chr.01	1012	14.8	14.6	425	41.1	96.6	1437	55.9	39.2
Chr.02	1853	26.2	14.1	261	25.3	97.1	2114	51.6	24.3
Chr.03	1160	18.8	16.2	212	28.9	136.1	1372	47.7	34.8
Chr.04	1249	18.8	15.0	390	30.1	77.3	1639	49.2	30.0
Chr.05	1425	21.8	15.3	83	18.8	227.1	1508	41.9	27.8
Chr.06	1326	22.0	16.6	315	28.6	90.8	1641	50.6	30.9
Chr.07	1630	27.5	16.9	168	17.0	101.4	1798	44.7	24.8
Chr.08	1960	30.5	15.6	182	15.8	86.5	2142	46.9	21.9
Chr.09	1262	17.4	13.8	369	29.2	79.0	1631	46.8	28.7
Chr.10	1536	24.1	15.7	337	26.7	79.2	1873	50.9	27.2
Chr.11	1241	24.3	19.6	111	14.7	132.2	1352	39.2	29.0
Chr.12	1140	17.0	14.9	135	22.6	167.2	1275	40.1	31.5
Chr.13	2086	29.5	14.1	171	14.7	86.2	2257	44.3	19.6
Chr.14	1345	20.3	15.1	307	29.3	95.3	1652	49.7	30.1
Chr.15	1779	22.9	12.9	309	27.5	89.1	2088	50.9	24.4
Chr.16	1246	17.6	14.1	225	19.6	87.3	1471	37.3	25.4
Chr.17	1422	20.1	14.2	289	21.6	74.7	1711	41.9	24.5
Chr.18	2480	36.6	14.7	309	25.5	82.6	2789	62.3	22.3
Chr.19	1660	27.4	16.5	331	23.2	70.0	1991	50.6	25.4
Chr.20	1007	17.6	17.5	261	26.6	101.9	1268	46.8	36.9
Average	1491	22.8	15.4	260	24.3	102.9	1750	47.5	27.9

### Population structure

Ln P (D) and ∆K were used to identify the number of subsets (K) in STRUCTURE. The analysis did not produce a clear ‘plateau’ as Ln P (D) increased gradually with the number of K (Additional file 5: Figure S3 A). However, the highest value of ∆K for the 166 soybean accessions was at K = 3 (Additional file 5: Figure S3 B). When K = 3, a total of 153 of the 166 accessions had a probability of greater than 0.6 of being in one of the three clusters (Additional file 5: Figure S3 C). Cluster 1 contained 15 accessions from Korea, Cluster 2 contained 39 accessions including 29 from China, 8 from Japan and 2 from Korea and Cluster 3 contained 112 accessions including 31 from China, 32 from Japan, and 49 from Korea. UPGMA dendrogram showed that clusters of the accessions were generally consistent with their geographic origins (Additional file 6: Figure S4).

### Fst estimation between the large and small seed weight sub-populations

**Fig. 1:**
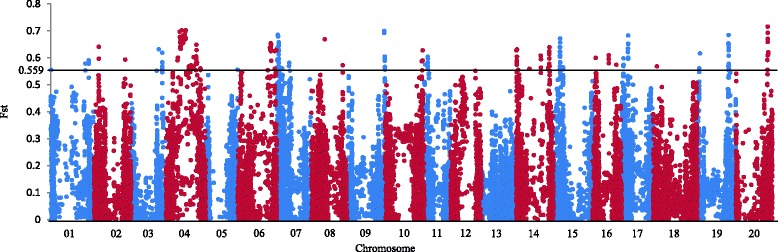
Manhattan plot of Fst. Manhattan plot of genome-wide Fst between large and small seed weight sub-populations against the position of SNPs on each of the 20 chromosomes

Genome-wide Fst was 0.145 between the large and small seed weight sub-populations with a standard deviation of 0.125, and the threshold of Fst was 0.557 at *P* = 0.001. Allele frequencies of 260 SNPs on 18 chromosomes were significantly different between the two sub-populations (Fig. 1 and Additional file 7: Table S3). Of the 260 SNPs, a total of 163 were in euchromatic regions and 97 in heterochromatic regions.

### Identification of QTL with large effect through GWAS and Fst analysis

**Fig. 2:**
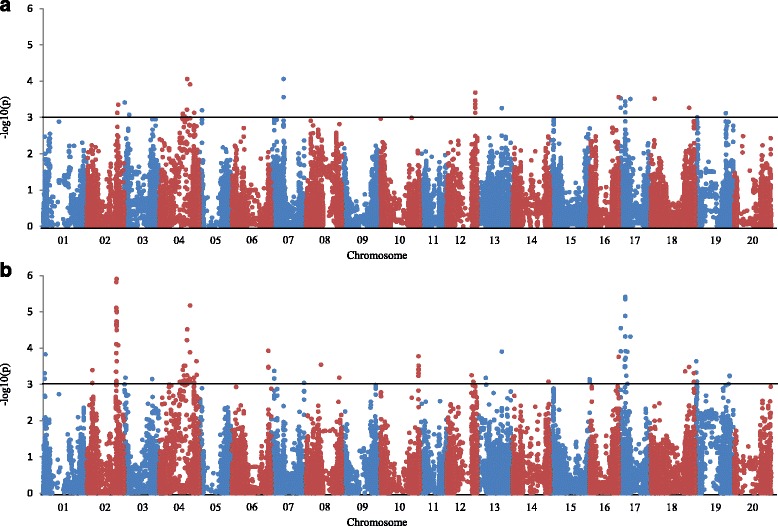
Manhattan plot of GWAS. Manhattan plot of GWAS of seed weight using MLM in the selective genotyping population grown at Beltsville, MD in 2012 (**a**) and 2013 (**b**)

Thirty-two SNPs across 11 of the 20 soybean chromosomes were associated with seed weight at –log (p) = 3.0 based on MLM association analysis for year 2012 (Fig. 2a). The phenotypic variation that was explained by each SNP varied from 6.68% to 9.62%. A total of 134 SNPs across 16 chromosomes were identified at –log (p) = 3.0 for year 2013 (Fig. 2b). The phenotypic variation explained by these SNPs ranged from 6.35% to 14.50%. Of the 17 SNPs across six chromosomes associated with seed weight in both years, eight had an Fst ≥ 0.557 between the large and small seed weight sub-populations (Table 2). The eight SNPs were considered as significant SNPs that were associated with large effect QTL. The seed weight difference between the accessions carrying alternative alleles for each of the eight SNPs ranged from 8.1 g to 11.7 g/100 seeds (*P* ≤ 0.001). For example, the mean 100-seed weight of the 96 accessions carrying the ‘T’ allele at the locus BARC_1.01_Gm04_37010886_T_C was 20.0 ± 6.70 g in 2012 and 22.2 ± 7.59 g in 2013. The mean seed weight of the 69 accessions with the alternative ‘C’ allele was 10.8 ± 3.75 g in 2012 and 11.3 ± 3.97 g in 2013 (Table 2 and Additional file 8: Table S4). With the exception of BARC_1.01_Gm04_37010886_T_C, the seven other SNPs were in three haplotype blocks (Table 3), i.e. four SNPs (BARC_1.01_Gm04_20336481_T_C, BARC_1.01_Gm04_20729591_G_A, BARC_1.01_Gm04_24276060_G_T, and BARC_1.01_Gm04_27912357_T_C) were in the haplotype block on chromosome 4 (haplotype block 1), two SNPs (BARC_1.01_Gm17_2500016_T_C and BARC_1.01_Gm17_2500333_T_G) were in the haplotype block on chromosome 17 (haplotype block 2), and one (BARC_1.01_Gm17_8635426_T_C) was in a haplotype block on chromosome 17 (haplotype block 3). Haplotype block 1 contained 145 SNPs and spaned about 15.2 Mbp in the heterochromatic region of chromosome 4 from 24.3 Mbp to 39.5 Mbp based on the Wm82a2v1 assembly [33, 34]. Haplotype block 2 contained 11 SNPs and spanned about 67.8 kbp in the euchromatic region of chromosome 17, while haplotype block 3 contained 13 SNPs and spanned about 168.7 kbp in the euchromatic region of chromosome 17. Four major haplotypes, each with more than ten individuals, were identified based on 145 SNPs in haplotype block 1. Seed weight was greater than 20.0 g for haplotype 2 and haplotype 4, but was less than 13.6 g for haplotype 1 and haplotype 3 in both years. There were three major haplotypes in both haplotype blocks 2 and 3. Significant differences in seed weight were also detected among different haplotypes in both haplotype blocks 2 and 3. Haplotype 2 in haplotype block 2, and haplotypes 1 and 3 in haplotype block 3 were associated with large seed weight.

**Table 2:** SNPs associated with seed weight identified by MLM based on 166 accessions grown at Beltsville, MD in 2012 and 2013

SNP ID	Allele	MAF	Fst	–log (p)		R^2^		Allelic effect (g)	
				2012	2013	2012	2013	2012	2013
Gm04_20336481	C:T	0.46	0.562	3.11	3.40	0.075	0.072	−3.99	−4.89
Gm04_20729591	A:G	0.46	0.569	3.01	3.09	0.069	0.073	−3.94	−4.64
Gm04_24276060	G:T	0.46	0.572	3.04	3.11	0.069	0.073	3.97	4.67
Gm04_27912357	C:T	0.43	0.602	4.06	4.52	0.096	0.111	−4.86	−6.00
Gm04_37010886	C:T	0.42	0.649	3.91	5.17	0.090	0.128	−5.22	−7.15
Gm17_2500016	C:T	0.28	0.574	3.27	3.91	0.073	0.092	−3.33	−4.27
Gm17_2500333	G:T	0.29	0.598	3.53	4.55	0.079	0.106	−3.51	−4.71
Gm17_8635426	C:T	0.41	0.635	3.33	5.41	0.075	0.132	−3.97	−6.15

**Table 3:** Haplotype (HAP) ID and position as well as haplotype effect on seed weight estimated based on 166 accessions grown at Beltsville, MD in 2012 and 2013, and on 3753 accessions from USDA Soybean Germplasm Collection at Germplasm Resources Information Network (GRIN)

SNP ID	HAP block			Seed weight for Hap within 166 accessions			Seed weight for Hap within 3753 accessions	
	HAP block ID	HAP block position	HAP name	Number of accessions	2012 (g)	2013 (g)	Number of accessions	Seed weight in GRIN (g)
Gm04_20336481	Block 1	24,300,386–39,511,809	HAP1	26	11.2	11.8	290	10.0
Gm04_20729591			HAP2	25	23.6	26.1	1104	20.7
Gm04_24276060			HAP3	17	13.2	13.6	181	17.0
Gm04_27912357			HAP4	14	20.0	23.2	121	19.4
Gm17_2500016	Block 2	2,485,630-2,553,448	HAP1	69	13.8	14.7	1435	14.2
Gm17_2500333			HAP2	44	23.2	25.9	1022	22.3
			HAP3	27	10.8	11.4	286	9.4
Gm17_8635426	Block 3	8,296,198-8,464,870	HAP1	68	19.9	22.2	2151	18.9
			HAP2	47	10.2	10.2	512	9.3
			HAP3	21	20.3	23.4	201	19.5

### Test of the haplotype association with seed weight using 3753 accessions from USDA soybean germplasm collection

The association of the SNPs with seed weight based on 166 accessions was further examined in a panel of 3753 accessions with 100-seed weight either greater than 20 g or smaller than 10 g as described for USDA Soybean Germplasm Collection at GRIN (http://www.ars-grin.gov/npgs/index.html#). Each of the eight significant SNPs detected in the analysis of the 166 accessions were also associated with seed weight in the 3753 accessions (Additional file 8: Table S4). For example, the ‘T’ and ‘C’ alleles of BARC_1.01_Gm04_20336481_T_C were associated with large and small seed weight in the 166 accessions, respectively, and among the 3753 accessions the 100-seed weight for the accessions carrying the ‘T’ allele was 19.0 ± 7.71 g and 11.4 ± 6.70 g for those with the ‘C’ allele. The haplotype contribution to the seed weight based on 3753 accessions was consistent with that based on 166 accessions (Table 3), e.g. a total of 290, 1104, 181 and 121 individuals were HAP1, HAP2, HAP3 and HAP4 in haplotype block 1, respectively, and their average 100-seed weight was 10.0 ± 5.49 g, 20.7 ± 7.39 g, 17.0 ± 7.21 g, and 19.4 ± 5.91 g, respectively.

### Correlation coefficient of Fst value with the proportion of variance explained and allelic effects of the SNPs

Permutation test showed that the correlation coefficient ranged from 0.411 to 0.680 and 0.388 to 0.649 between Fst and R^2^, and between Fst and absolute allelic effects among the 20 sets of random samples, respectively. The Fst is also significantly associated with the LOD value of the GWAS with an average correlation coefficient of 0.557 (*P* < 0.01).

## Discussion

In this study, we conducted GWAS to detect QTL associated with seed weight in soybean using selective populations. The mapping panel was selected from accessions of Maturity Groups II, III and IV. These Maturity Groups were chosen because of their photoperiod response allows them to mature appropriately in field trials in Beltsville, Maryland region. The purpose was to minimize the collection of inaccurate phenotypic data due to poor adaptability of the accessions that were not well suited to the growing region. Synthetic association due to genetic heterogeneity is one of the major limitations of GWAS. This is despite the fact that the MLM in GWAS takes into account both population structure and relative kinship [35], which can greatly reduce the false positive rate in GWAS compared with the general linear model that considers population structure only, and the simple model which doesn’t take into account population structure or relative kinship [1, 5, 8]. In this study, accessions with similar maturity were chosen in order to reduce the genetic heterogeneity and issues related to normal growth and maturity, as maturity is related to the genetic structure in soybean [22]. In addition, the mapping panels were carefully balanced in terms of country of origin, maturity group and growth habit.

The power of GWAS to detect the association between SNPs and traits depends on the percentage of phenotypic variance that can be explained by markers, while the phenotypic variance is determined by allelic effects, and the allele frequency in the panel [36, 37]. In previous soybean seed GWAS, most association panels may have had a low frequency of the genotypes containing causative alleles. In this study, by increasing the frequency of the accessions with large and small seed weight, the frequency of variants with pronounced effect on seed weight was expected to be elevated within the panel, thus the major effects could be detected. These variants would otherwise be rare in random populations and might not be detected via genome-wide association analysis. Zhang et al. [1] performed GWAS of seed weight using a set of 309 random accessions. These accessions were genotyped with the SoySNP50K BeadChip and analyzed with the MLM method. The frequency of accessions with large (>20 g) or small seed weight (<10 g) was less than 10% based on the frequency distribution of mean seed weights of the 309 accessions over 4 environments. The MAF of the significant SNPs ranged from 0.05 to 0.43 and the allelic effects ranged from 0.43 g to 1.29 g/100 seeds. However, in the present study, the MAF and allelic effects varied from 0.28 to 0.46 and 3.33 g/100 seeds to 7.15 g/100 seeds, respectively. The average of both parameters was higher in the present study than in Zhang’s report [1]. Also, the variation explained by significant markers (R^2^) ranged from 0.069 to 0.132, and was higher than that reported by Zhang et al. [1] (from 0.018 to 0.038) and others [8, 9]. Using selective genotypes as a mapping panel could also facilitate cross-examination of QTL from GWAS by the Fst test. The Fst method has been used to identify genomic regions associated with domestication and selective sweeps associated with breeding in soybean [38–40]. By taking advantage of selective populations, we also calculated Fst values of the SNPs between the accessions with large and small seed weight. In addition, this further validates the results from GWAS analysis. Permutation test verified that the Fst values are significantly associated with the R^2^ and allelic effects among the 20 sets of random samples. In addition, we also observed that the average 100-seed weight differences between the two alleles of the SNPs significant in both Fst test and GWAS vs. GWAS alone ranged from 8.9 to 11.2 and 0.51 to 8.7 g in two years, respectively (Additional file 8: Table S4). The average seed weight difference between the two alleles of the SNPs significant in both Fst test and GWAS is on average 3.6 g bigger than that of the SNPs significant in GWAS only. Similar result was also observed based on the seed weight of 3753 accessions in the germplasm, the average seed weight difference between the two alleles of the SNPs which were significant in both Fst test and GWAS of the 166 accessions is on average 3.2 g bigger than that of the SNPs significant in GWAS only (Additional file 8: Table S4). The observation suggested that the Fst with GWAS analysis were more likely to identify the SNPs with profound effects than the GWAS only.

Although more than 200 QTL associated with seed weight have been identified in the past decades [41], most had a small effect on seed weight because most linkage mapping populations used in these studies were created from parents with a small difference in seed weight. For example, the additive effect of the QTL Sd_wt 36–15 mapped on chromosome 4 [42] was only 0.2 g as the seed weight difference between the two parents was only 3.7 g over 3 years. In the present study, the corresponding region (haplotype block 1 of chromosome 4) showed additive values varying from 3.94 to 6.00 g. We also identified major QTL in two haplotype blocks on chromosome 17, one was in the interval from 2,485,630 bp to 2,553,448 bp, and another in the interval from 8,296,198 bp to 8,464,870 bp, explaining 13.2% and 6.0 g of the phenotypic variation and additive effects, respectively. QTL in this region of chromosome 17 were reported in linkage studies of RIL populations. Of the three QTL identified in the ‘Kefeng No. 1’ × ‘Nannong 1138–2’ population, the QTL in the region on chromosome 17 explained about 11.4% of phenotypic variance with LOD of 4.8 and an additive effect of 0.6 g [43]. The LOD, R^2^ and additive effect of this QTL were all larger than those of the other two QTL. This QTL also had the second largest R^2^ among six QTL that were identified in the RIL population of ‘A97–775019’ × ‘A96–492041’ by Hoeck et al. [44].

One suggested virtue of association studies is the ability to take advantage of existing phenotypes. However, Hwang et al. [5] reported that there were some difficulties in performing association studies of soybean seed protein and oil content using the existing phenotypic data deposited at the GRIN since the traits were measured in different years and locations. In this study, the correlation of seed weight measured at Beltsville, Maryland for two years and the data reported in the GRIN were highly significant (*r* = 0.95). The designation of the common accessions to large and small seed weight groups based on USDA Soybean Germplasm Collection data, was consistent with that based on seed weight observed in 2012 and 2013, even though the GRIN data were obtained in different years and/or environments and variation of seed weight due to genetic environmental interaction was expected. Thus, the seed weight from the 3753 accessions of USDA Soybean Germplasm Collection was successfully used to verify its association with SNPs and haplotypes.

Identifying candidate genes controlling traits is a challenge in genetic research. In this study, QTL associated with seed weight were identified in three haplotype blocks and an independent SNP locus. A survey of the soybean genome indicated 212 genes in haplotype block 1, 10 in the haplotype block 2, and 21 in the haplotype block 3, however, we didn’t find a homologous gene in soybean that was related to seed weight in other species [45, 46], except for Glyma.04G143300 in haplotype block 1. The gene function of Glyma.04G143300 was predicted as an AP2/B3-like transcriptional factor family protein [33] which was related to seed weight in *Arabidopsis* [46]. It is possible that soybean genes controlling seed weight may be structurally different than the genes in other species or that we still know very little about the nature of seed weight genes.

## Conclusion

A selective population consisting of 166 large or small seed weight soybean accessions was used to detect QTL with pronounced effects on seed weight. Based on the association analysis of the seed weight observed in two years with 35,009 SNP markers as well as the analysis of SNP allelic difference between the large and small seed sub-populations, we observed SNPs and haplotypes in three haplotype blocks on chromosomes 4 and 17 that are related to the major QTL/genes contributing to the seed weight variation. The average difference of 100-seed weight between the accessions with two different alleles among the significant SNPs varied from 8.1 g to 11.7 g. The significant association of the haplotypes with seed weight was further validated in a panel with 3753 accessions of the USDA Soybean Germplasm Collection. This is the first report that attempts to identify major QTL controlling seed weight in soybean using selective genotyping GWAS and fixation index analysis. The results and methods described here will assist us to efficiently identify major genes controlling seed weight and to fully understand the genetic mechanisms underlying seed weight variation. This approach may also help geneticists and breeders to more efficiently identify major QTL controlling other traits.

## Additional files

Additional file 1: Table S1. PI entries and their country of origin, maturity group, 100-seed weight, and other traits reported by the Germplasm Resources Information Network (GRIN). (XLSX 25 kb)

Additional file 2: Figure S1. The phenotypic distribution of seed weight for accessions grown at Beltsville, MD in 2012 and 2013. (PDF 88 kb)

Additional file 3: Table S2. Analysis of variance for seed weight of the 166 accessions grown at Beltsville, MD in 2012 and 2013. (XLSX 9 kb)

Additional file 4: Figure S2. Extent of linkage disequilibrium (LD) in euchromatic (black) and heterochromatic (gray) regions of the 20 soybean chromosomes. (PDF 219 kb)

Additional file 5: Figure S3. Population structure inferred by Bayesian clustering approaches based on SNPs and the schematic clustering procedure from STRUCTURE. Plots of cluster number vs. mean LnP(D) (A) and ∆K (B) over 5 runs for each K value. Three clusters were inferred (C). (PDF 29 kb)

Additional file 6: Figure S4. Genetic relationship of 166 PIs from Korea, Japan and China. Genetic relationship was based on the unweighted pair group with arithmetic mean (UPGMA) method. (PDF 20 kb)

Additional file 7: Table S3. Loci with Fst value >0.557 between the large and small seed weight populations across the soybean genome. (XLSX 24 kb)

Additional file 8: Table S4. Seed weight difference for SNPs associated with seed weight based on 166 accessions grown at Beltsville, MD in 2012 and 2013, and 3753 accessions from USDA Soybean Germplasm Collection. (XLSX 14 kb)

## Abbreviations

Fst: Fixation index
GRIN: Germplasm Resources Information Network
GWAS: Genome-wide association study
LD: Linkage disequilibrium
MAF: Minor allele frequency
MLM: Mixed linear model
PI: Plant introduction
QTL: Quantitative trait loci
RIL: Recombinant inbred line
SNP: Single nucleotide polymorphism
UPGMA: Unweighted pair group with arithmetic mean
