# Supplementary figures and images for: Identification of QTL with large effect on seed weight in a selective population of soybean with genome-wide association and fixation index analyses
Source: BMC Genomics. 2017 Jul 12;18:529. doi: 10.1186/s12864-017-3922-0 (PMC5508781; doi:10.1186/s12864-017-3922-0)

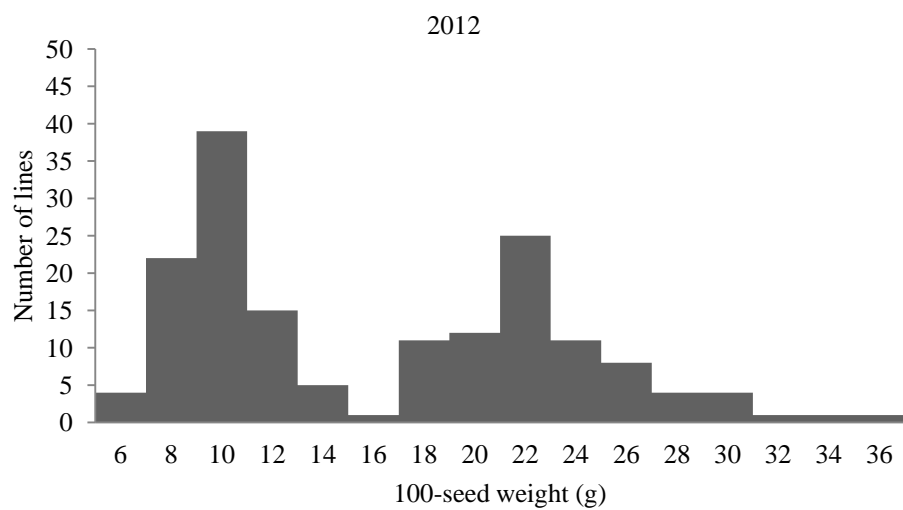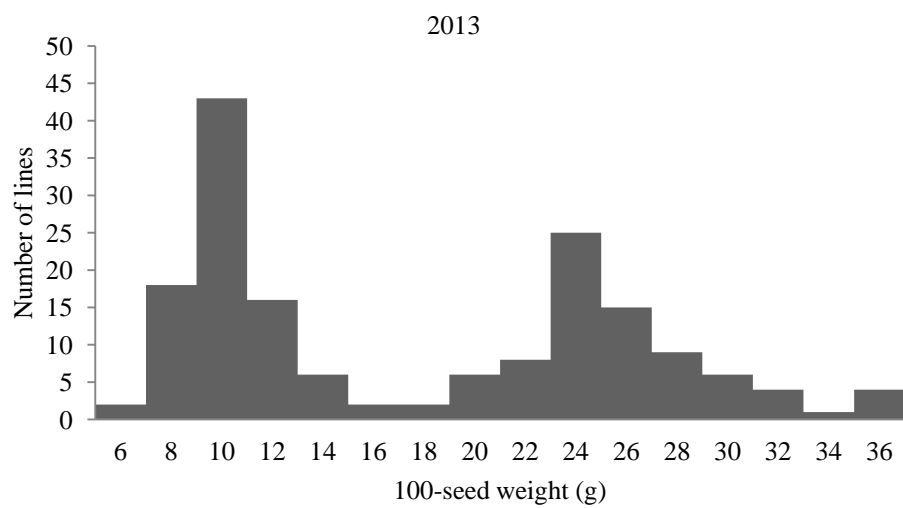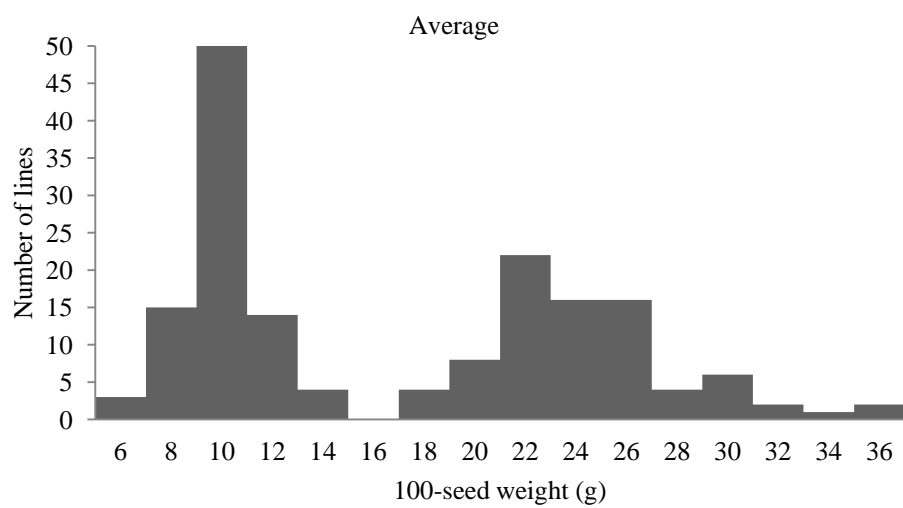

Supplement: Supplementary file 2 — The phenotypic distribution of seed weight for accessions grown at Beltsville, MD in 2012 and 2013. (PDF 88 kb) [file 12864_2017_3922_MOESM2_ESM.pdf]

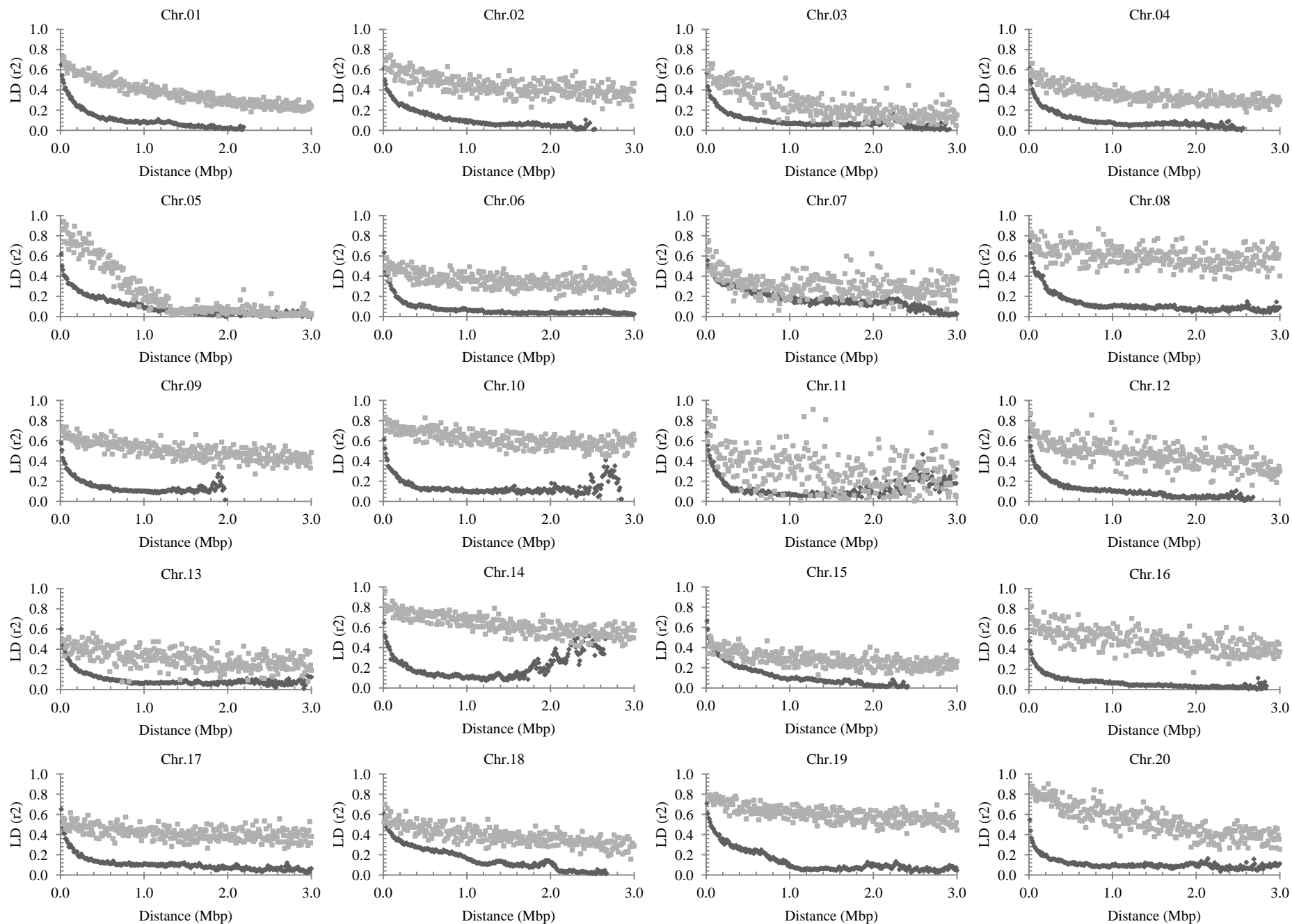

Supplement: Supplementary file 4 — Extent of linkage disequilibrium (LD) in euchromatic (black) and heterochromatic (gray) regions of the 20 soybean chromosomes. (PDF 219 kb) [file 12864_2017_3922_MOESM4_ESM.pdf]

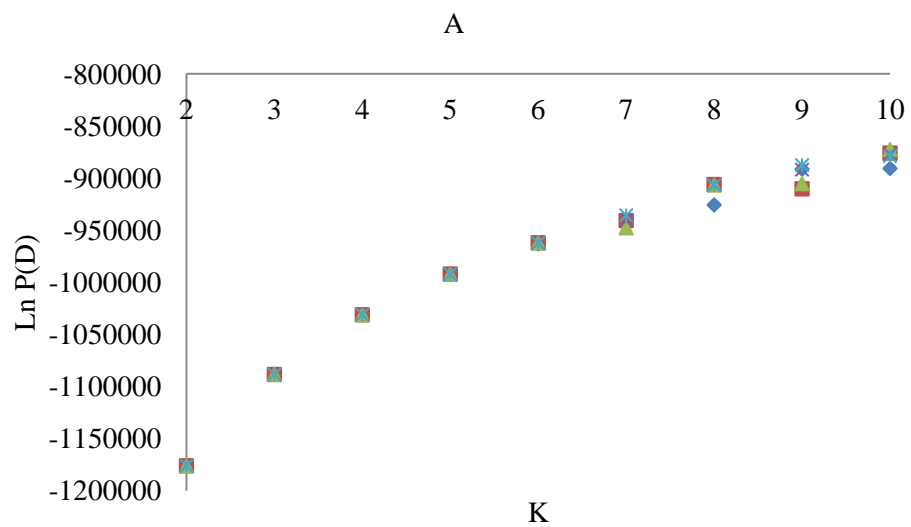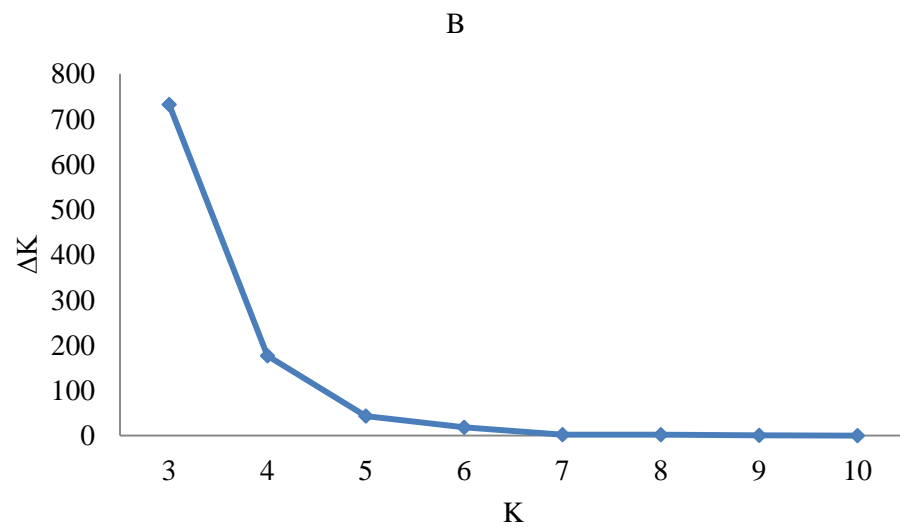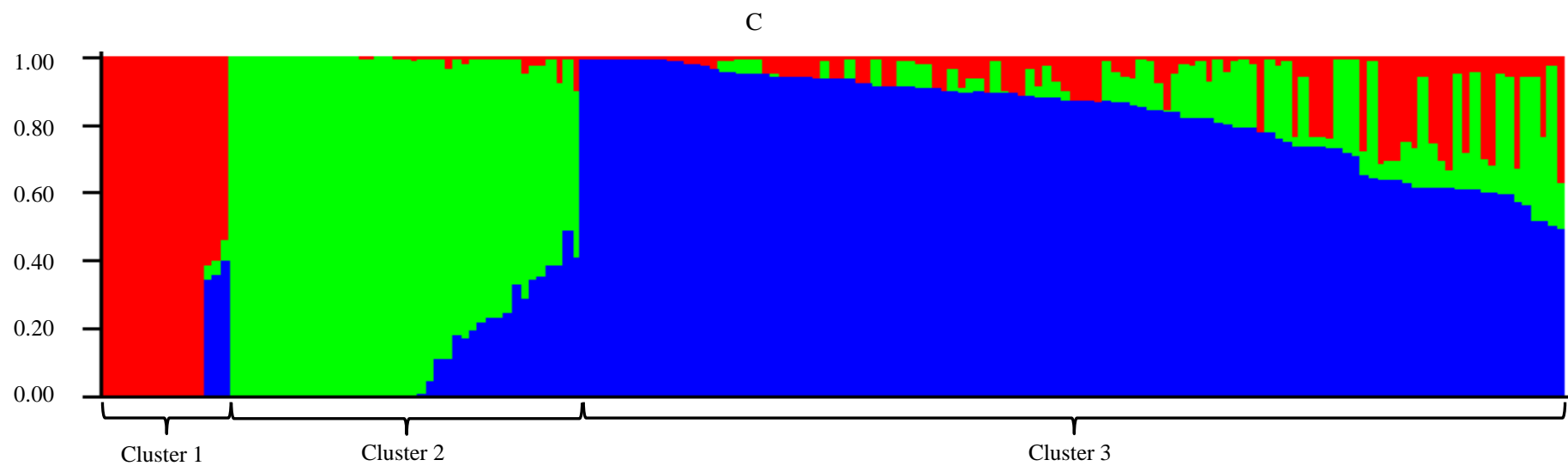

Supplement: Supplementary file 5 — Population structure inferred by Bayesian clustering approaches based on SNPs and the schematic clustering procedure from STRUCTURE. Plots of cluster number vs. mean LnP(D) (A) and ∆K (B) over 5 runs for each K value. Three clusters were inferred (C). (PDF 29 kb) [file 12864_2017_3922_MOESM5_ESM.pdf]

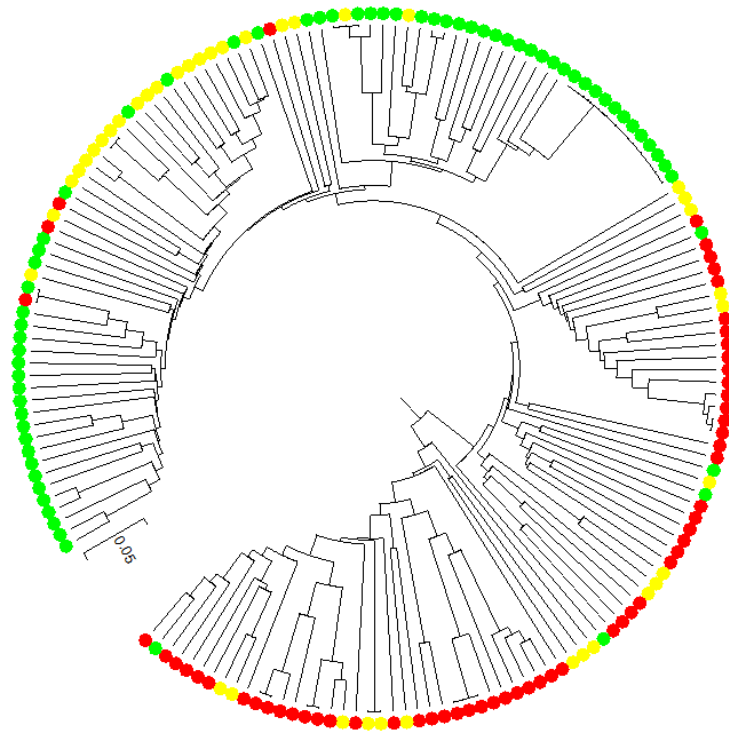

- Korea
- Japan
- China

Supplement: Supplementary file 6 — Genetic relationship of 166 PIs from Korea, Japan and China. Genetic relationship was based on the unweighted pair group with arithmetic mean (UPGMA) method. (PDF 20 kb) [file 12864_2017_3922_MOESM6_ESM.pdf]
